# Genetically confirmed severe hemophilia A in a preterm infant: a case report

**DOI:** 10.3389/fped.2026.1837559

**Published:** 2026-09-14

**Authors:** Lanjun Mu, Chan Wang, Yuqian Wang, Jing Zhu, Li Zhang, Ning Zou

**Affiliations:** Department of Pediatrics, The Second Hospital of Dalian Medical University, Dalian, Liaoning Province, China

**Keywords:** coagulation factor VIII, emicizumab, neonatal hemorrhage, preterm infant, severe hemophilia A

## Abstract

Severe hemophilia A is exceptionally rare among preterm infants. Qwing to immature coagulation system and nonspecific bleeding manifestations, the condition is readily misdiagnosed as other neonatal hemorrhagic disorders, such as vitamin K deficiency, neonatal sepsis and thrombocytopenia. Delayed diagnosis will greatly increase the risk of severe complications, particularly intracranial hemorrhage. Herein, we report a case of genetically confirmed severe hemophilia A in a 34-week preterm infant without family history of hemophilia. Treatment comprised sequential adminnistration of coagulation factor VIII repalcement and emicizumab. We futher analyze the clinical characteristics, diagnostic difficulties and standardized management principles of hemophilia A in preterm infants, so as to improve clinicians' awareness, reduce misdiagnosis and missed diagnosis, and optimize the long-term prognosis of affected infants.

## 1 Introduction

Hemophilia A (HA) is an inherited hemorrhagic disorder caused by variants in the gene encoding coagulation factor VIII (FVIII), leading to deficient or dysfunctional factor VIII. It is clinically manifested as spontaneous bleeding or persistent bleeding following minor trauma (1). Among live-born males, the birth prevalence of hemophilia A is approximately 24.6 per 100,000, with severe hemophilia A accounting for 9.5 per 100,000, representing a relatively rare clinical entity (2). Preterm infants exhibit an immature coagulation system, atypical bleeding manifestations, reduced levels of multiple coagulation factors, and impaired platelet function. In thoses with an unremarkable family history or only mild bleeding symptoms, clinicians tend to prioritize common neonatal disorders such as vitamin K₁ deficiency and neonatal sepsis. This often results in misdiagnosis and increases the risk of life-threatening hemorrhage, particularly intracranial hemorrhage (3, 4).

Most previously reported cases of hemophilia A in preterm infants have involved extremely preterm infants born at 26–28 weeks of gestation, some of whom had delayed diagnosis, comorbidities or a positive family history. In contrast, our patient was a preterm infant at 34 weeks of gestation with no family history of hemophilia and genetic testing comfirmed the diagnosis during the neonatal period. The infant received sequential therapy with factor VIII and emicizumab during hospitalization, and no life-threatening complications such as intracranial hemorrhage occurred. The full clinical course documented herein makes this case a valuable clinical reference.

In preterm infants, conventional factor VIII replacement therapy is limited by difficult venous access, short half-life and a high risk of inhibitor development (5). Emicizumab, a subcutaneously administered bispecific monoclonal antibody, significantly improves treatment adherence and long-term therapeutic outcomes (5). Nevertheless, pharmacodynamic and pharmacokinetic data regarding this agent in neonates and preterm infants remain limited.

Accordingly, we retrospectively describe the clinical course of the patient, summarize practical experience and pitfalls, and discuss the clinical features as well as diagnostic and therapeutic essentials of hemophilia A in preterm infants, with the aim of improving clinicians' proficiency in recognizing and managing this condition.

## 2 Case presentation

### 2.1 Clinical characteristics

The patient was a male preterm infant born at 34 weeks of gestational age. The mother, gravida 2, para 1, had a preterm delivery attributed to intrahepatic cholestasis of pregnancy and bilateral ovarian enlargement (suspected ovarian hyperstimulation syndrome). The infant was delivered by cesarean section at our hospital. At birth, he had a single nuchal cord, with normal amniotic fluid and placenta. Birth weight was 2,200 g. Apgar score was 9 points at 1 min (1 point deducted for respiration), and 10 at 5 and 10 min. The mother experienced no active bleeding during cesarean delivery and was discharged on postoperative day 3; her factor VIII activity was not assessed. The infant subsequently developed progressive respiratory distress and was admitted to our neonatal intensive care unit (NICU). The parents were non-consanguineous and denied any history of hereditary diseases. The mother received regular prenatal examinations throughout pregnancy and had no hematological disorders.

Physical examination on admission: He was conscious and responsive, with no dysmorphic facial features. The skin was pale, swollen and erythematous, accompanied by increased tension and fissures in the skin folds (Figures 1A,B). The anterior fontanelle was flat and soft. Nasal flaring and respiratory distress were present. Coarse breath sounds and moist rales were heard bilaterally in the lungs. The umbilical cord was intact without bleeding or exudation. No obvious abnormalities were found in cardiovascular, abdominal, neurological, or other systemic examinations (Figure 2).

**Figure 1:**
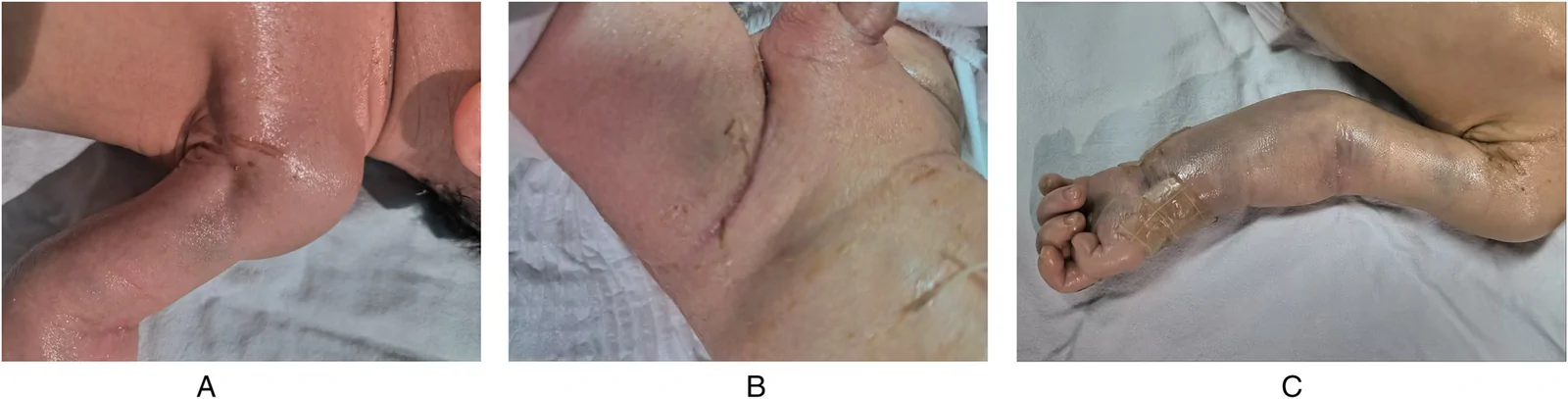
Clinical signs of the preterm infant (written informed consent was obtained from the parents for publication of the clinical images. **(A, B)**: The skin was pale, swollen and erythematous, accompanied by increased tension and fissures in the skin folds. **(C)**: Edema, ecchymosis and indurated swelling were noted on the left forearm.

**Figure 2:**
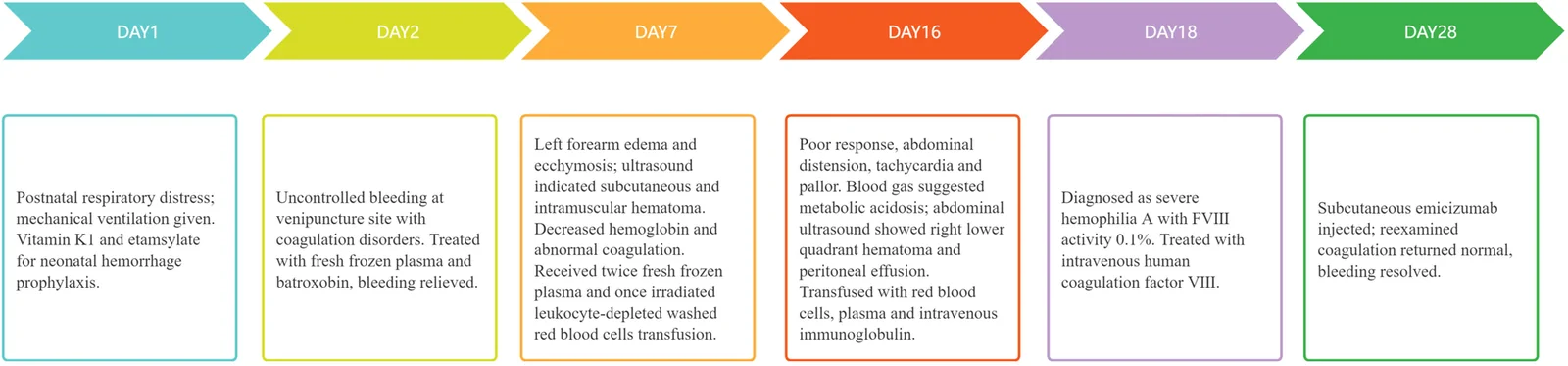
Clinical Diagnosis and Clinical Course.

### 2.2 Clinical course and interventions

Day 1 after admission, the infant received mechanical ventilation, antimicrobial therapy, and nutritional support. Intravenous vitamin K1 and ethamsylate were administered for the prevention of neonatal hemorrhagic disease.

Day 2 after admission: The infant developed persistent oozing from venipuncture site taht was refractory to local copression. Emergency laboratory tests showed a C-reactive protein level of 0.21 mg/L, a white blood cell count of 8.55 × 10⁹/L, a hemoglobin level of 199 g/L,and a platelet count of 224 × 10⁹/L. Blood gas analysis, liver and renal function, thyroid function, electrolytes, urine and stool routine, sputum culture, and blood culture were all unremarkable. Imaging studies showed bilateral choroid plexus cysts on cranial ultrasound, hydrocele on testicular ultrasound, and patent foramen ovale (diameter: 3.2mm) on cardiac ultrasound. Coagulation profile revealed prothrombin time (PT) 14.9 s (reference range: 10–25s); activated partial thromboplastin time (APTT) 135.6 s(reference range: 25–68s); thrombin time (TT) 21.2 s(reference range: 11–17s); fibrinogen (Fib) 1.52 g/L(reference range: 1.5–3.25 g/L); D-dimer 0.87 mg/L(reference range: 0–0.5 g/L) (instrument-based one-stage clotting assay); Bleeding improved after supportive hemostatic therapy with transfusion of fresh frozen plasma (FFP) and hemocoagulase. Considering the abnormal skin manifestations after birth, congenital ectodermal dysplasia and ichthyosis were suspected, and genetic testing was performed for differential diagnosis.

Day 7 after admission: Edema, ecchymosis and indurated swelling were noted on the left forearm (Figure 1C). Ultrasonography confirmed a hematoma in the subcutaneous and muscular layers. Repeat coagulation tests revealed a PT of 14.8 s, an APTT of 140.6 s,a TT of 22.8 s, and a Fib of 0.73 g/L. The infant received two units of FFP transfusion. Due to progressive anemia, irradiated leukoreduced washed red blood cells were transfused, and the hematoma gradually resolved.

Day 16 after admission: The infant acutely developed poor responsiveness, abdominal distension, tachycardia, and pallor. Coagulation tests showed a PT of 14.6 s; an APTT of 132.3 s; a TT of 15.3 s; and a Fib 3.4g/L. Blood gas analysis indicated metabolic acidosis. Abdominal ultrasound revealed a right lower quadrant hematoma and peritoneal effusion. Immediate treatment included hemocoagulase agkistrodon, ethamsylate, vitamin K1, transfusion of red blood cells, FFP, and intravenous immunoglobulin, with intensified antimicrobial therapy.

Day 18 after admission: The results of coagulation factor activity assays were obtained: factor VIII activity 0.1%; factor IX activity 29.7%; factor X activity 49.1%; factor XII activity 13.8%; von Willebrand factor (vWF) 216.7%(instrument-based one-stage clotting assay); the plasma inhibitor titer against factor VIII was less than 0.6 BU/mL. Combined with clinical manifestations and laboratory findings, a definitive diagnosis of severe hemophilia A was established. The infant received intravenous human coagulation factor VIII (standard half-life factor concentrates)at a dose of 40 IU/kg. Factor VIII activity was monitored at 15 min, 3 h, 24 h, and 65 h after infusion, with corresponding levels of 117.6%, 92.5%, 11.1% and 0.5%, respectively. The dose was subsequently adjusted to 50 IU/kg and continued for 5 infusions.

Day 28 after admission: Factor VIII replacement had effectively controlled the bleeding. No active bleeding was observed,and anemia was corrected. The hematomas at the puncture site and left forearm gradually resolved, and the abdominal hematoma showed remarkable absorption. Due to challenging venous access and an increased risk of inhibitor development associated with conventional factor VIII therapy, the infant received a single subcutaneous dose of emicizumab 3 mg/kg before discharge with parental informed consent for long-term bleeding control.

No severe adverse events, including intracranial hemorrhage or inhibitor development, occurred during hospitalization. Long-term prevention with emicizumab was scheduled after discharge, but the infant's family chose to continue follow-up at a higher-level specialized hemophilia hospital. Due to loss to follow-up, we were unable to collect long-term outcome data and evaluate the long-term therapeutic efficacy in this case.

### 2.3 Genetic testing

Whole-exome sequencing of peripheral blood samples from the infant and his parents identified a hemizygous c.6202A > T (p.Lys2068Ter) variant in the F8 gene (Table 1). This nonsense variant is located in the coding region and results from an adenine-to-thymine substitution at nucleotide position 6,202 which conxerts the lysine at amino acid position 2,068 into a premature termination codon and leads to truncated protein synthesis. Segregation analysis revealed taht the mother was a heterozygous carrier of the variant, whereas the fathere habored the wild-type alleie at this locus (Figure 3). According to the criteria established by the American College of Medical Genetics and Genomics (ACMG), this variant was preliminarily classified as a variant of likely pathogenic significance.

**Figure 3:**
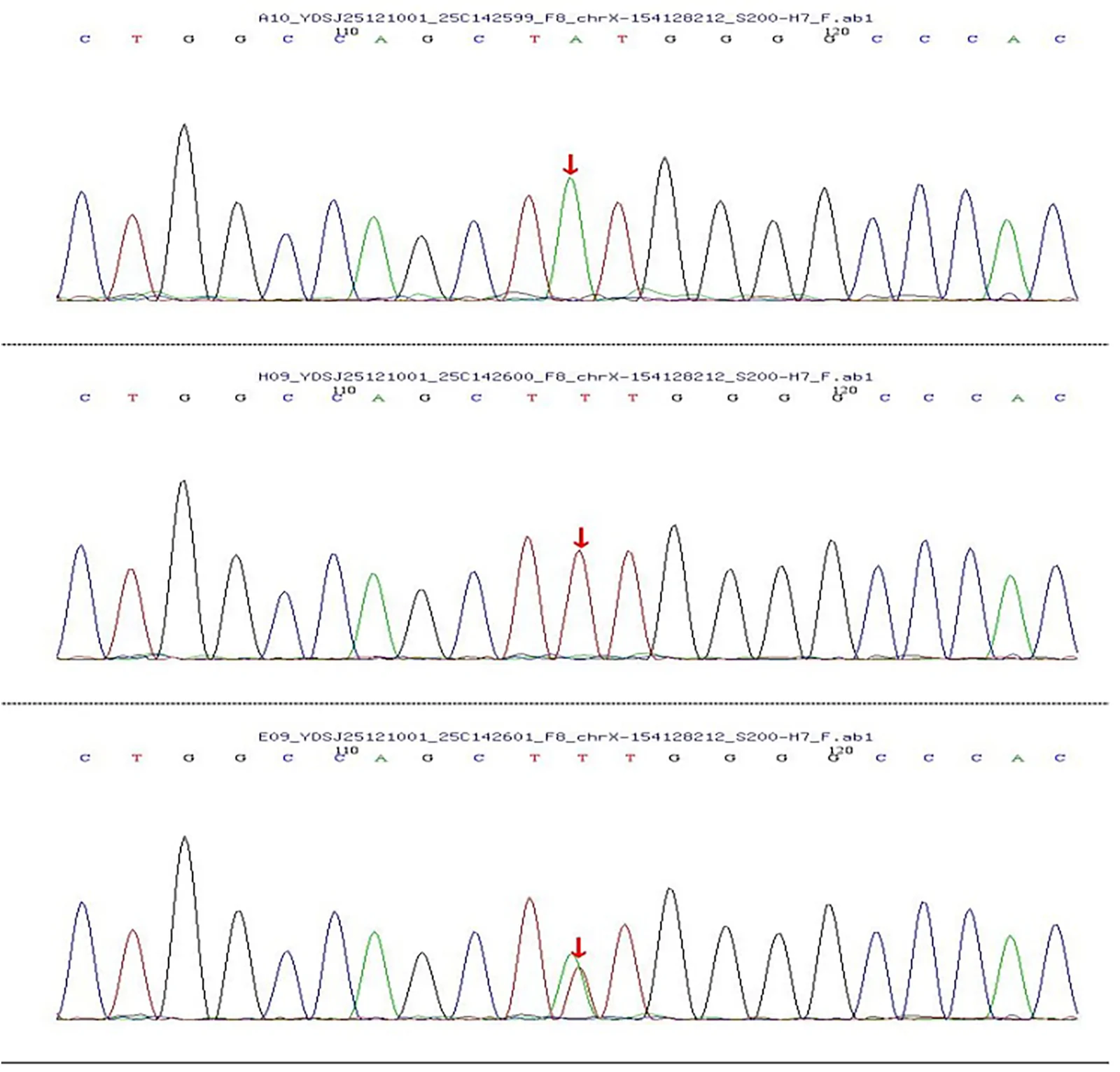
Sanger sequencing electropherograms of the F8 gene in the infant and his family members. The upper electropherogram indicated a hemizygous mutation of c.6202A > T (p.Lys2068Ter) in the F8 gene of the infant; the middle one demonstrated no genetic variation at the corresponding locus in the father; the lower one revealed a heterozygous variation at the identical locus in the mother.

**Table 1:** F8 gene detection results of the preterm infant with severe hemophilia A.

Gene	Chromosomal Location	Transcript & Exon	Nucleotide and Amino Acid Mutation	Variation Type
F8	ChrX: 154128212	NM_000132.4; Exon 21	c.6202A > T (p.Lys2068Ter)	Hemizygous mutation

## 3 Discussion

Hemophilia A is a hemorrhagic disorder caused by deficiency or reduced activity of coagulation factor VIII (FVIII) (1). It is an X-linked recessive disorder, which predominantly affects males while females usually act as carriers (6). Approximately one-third of cases are sporadic with no positive family history, mostly resulting from *de novo* mutations. According to FVIII activity levels, hemophilia A is classified as mild (5%–40%), moderate (1%–5%), or severe (<1%) (7). Unlike older children, who predominantly present with spontaneous joint and muscle bleeding, neonates typically manifest as persistent bleeding at puncture sites, umbilical stump oozing, subcutaneous ecchymoses, cephalohematoma, and intracranial hemorrhage (8).

In this present case, the 34-week preterm infant had no family history of hereditary disease and received routine vitamin K1 and ethamsylate for hemorrhage prophylaxis after birth. Skin abnormalities observed early in the clinical course prompted initial consideration of ectodermal dysplasia-associated disorders. Subsequently, the infant developed recurrent bleeding fron puncture sites, as well as limb and abdominal hematomas. Clinically, acquired coagulation disorders are often considered first, among which disseminated intravascular coagulation is the most common. However, this infant exhibited a markedly prolonged APTT but normal platelets in multiple monitorings, and recurrent bleeding symptoms, even abdominal hematoma, despite plasma transfusion. A lack of clinical awareness delayed timely measurement of FVIII activity, which in turn postponed the diagnosis. For preterm infants with unexplained spontaneous, persistent, or severe bleeding, basic coagulation tests including complete blood count, APTT, PT, TT, and fibrinogen should be performed as soon as possible, and FVIII activity should be determined early (9). In addition, detailed family genetic history should be obtained to assess carrier risk, and genetic testing should be conducted for highly suspected cases to confirm the diagnosis (10). For infants with a positive family history, collection of umbilical cord blood at delivery for factor VIII activity testing and genotyping enables early diagnosis of hemophilia A in the neonatal period.

The cornerstone of hemophilia A management comprises bleeding prophylaxis, local hemostatic measures, and coagulation factor VIII replacement therapy. Once the diagnosis is confirmed, bleeding control should be implemented following the principles of early initiation, adequate dosing, and sufficient duration. In cases of suspected intracranial hemorrhage, FVIII replacement therapy must be initiated immediately without delay for imaging studies (11). For severe cases, transfusion of fresh frozen plasma is indicated as an adjunct to rapidly restore circulating coagulation factor levels.

In this case, bleeding was promptly controlled and hematomas resolved completely following FVIII replacement, with no notable adverse events. However, high dose or frequent FVIII infusion is associated with an increased risk of inhibitor development, necessitating careful titration of dose and infusion frequency (12, 13). Notably, this infant harbored a nonsense mutation in the F8 gene, a genotype that has been correlated with a higher incidence of high-titer inhibitors and higher peak inhibitor titers (14). This genotypes substantially elevate bleeding risk, complicate clinical management, increase therapeutic costs, and may worsen mortality and long-term quality of life.

Emicizumab (EMI), a recombinant bispecific monoclonal antibody, binds activated factor IX and factor X, thereby mimicking the cofactor function of activated FVIII (15). Its efficacy is not affected by FVIII inhibitors, making it a favorable therapeutic option for patients with congenital or acquired FVIII inhibitors complicating hemophilia A (16, 17). Administered subcutaneously, emicizumab has a half-life of 28–30 days, which markedly improves treatment adherence and renders it suitable for long-term prophylaxis in neonates and preterm infants (18, 19). Adverse events are mostly mild-to-moderate, including injection-site reactions, headache, and upper respiratory tract infection, and generally resolve spontaneously. Caution is warranted when activated prothrombin complex concentrate, as this combination may increase the risk of thromboembolism and thrombotic microangiopathy; the drug should be discontinued immediately if such complications occur (20). In the present infant, satisfactory hemostasis was achieved with FVIII replacement and a single subcutaneous dose of emicizumab 3 mg/kg was administered before discharge. Long-term efficacy could not be evaluated, because of loss to follow-up. Further large-scale, multicenter studies are needed to clarify the optimal dosing, long-term efficacy and safety of emicizumab in preterm infants, to refine standardized screening, diagnosis, and treatment protocols for rare hemorrhagic disorders in preterm infants, and to reduce rates of missed diagnosis and misdiagnosis, ultimately improve long-term prognosis.

We report a sporadic case of severe hemophilia A in a preterm infant born at 34 weeks of gestation who lacked any hereditary background of hemophilia. Coagulation tests, factor activity assays and whole-exome sequencing were completed during admission, which confirmed the underlying pathogenic mutation. Early treatment with factor VIII preparations and the novel drug emicizumab was administered successively. The detailed diagnosis and treatment process described in this case provides valuable practical experience for junior medical staff and clinicians working at primary medical institutions.

Severe hemophilia A presents with non-specific manifestations in preterm infants. Early recognition of bleeding symptoms is crucial for timely diagnosis and intervention. A delay in diagnosis may lead to clinical deterioration and life-threatening comlications, while also gennerating additional costs from unnecessary investigations for recurrent bleedding and repeated hospitalizations for hemostayic therapy, therby imposing a substantial financial burden on the family. When unexplained recurrent or persistent bleeding, skin hematoma, intracranial hemorrhage and other symptoms occur, hemophilia should be considered, and coagulation factor detection and genetic testing should be actively performed. In this case, genetic testing established the etiological diagnosis, which not only facilitates screening for high-risk gene variants predisposing to inhibitor development and prediction of disease severity, thereby guiding individualized treatment, but also provides an essential basis for genetic counseling and prenatal screening should the infant's parents consider future pregnancies.

### 3.1 Limitations

In this case, insufficient clinician awareness of and experience with hemophilia A in preterm infants delayed coagulation factor teseing, management was guided solely by the prolonged APTT on routine coagulation studies, resulting in a delayed diagnosis and a missed window for optimal intervention. Furthermore, the infant was lost to follow-up after discharge, so it was impossible to evaluate the long-term efficacy of emicizumab, the recurrence of bleeding, and the growth and development outcomes, making it difficult to comprehensively evaluate the effectiveness and safety of the treatment plan.

## Data Availability

The original contributions presented in the study are included in the article/Supplementary Material, further inquiries can be directed to the corresponding author.
